# Case study: The DBT implementation journey of dialectical behaviour therapy in five European countries

**DOI:** 10.1186/s40479-026-00359-x

**Published:** 2026-07-14

**Authors:** Amy Gaglia Essletzbichler, Miriam Bierman, Magdalena Skuza, Natálie Češková, Anita Johanna Tørmoen

**Affiliations:** 1 Klinik für Psychosomatik und Psychotherapeutische Medizin Zentralinstitut für Seelische Gesundheit, J 5 68159 Mannheim, Germany; 2 National Center for Suicide Research and Prevention, University of Oslo, Oslo, Norway; 3 Veřejné duševní zdraví, VP3, National Institute of Mental Health (NIMH), Topolová 748, 250 67 Klecany, Prague, Czechia; 4 Centrum Zdrowia Psychicznego HarmonJa, Polish society for DBT, Gdansk, Poland; 5 Bangor, North Wales, UK

**Keywords:** DBT, Implementation, Case study

## Abstract

**Background:**

This paper is a narrative review of the implementation trajectories Dialectical Behaviour Therapy in five countries, the UK, Germany Norway, Poland, and the Czech Republic in Europe over the past 30 years. The aim of this comparison is to identify common and divergent systems levels facilitators and hindrances of DBT implementation.

**Methods:**

DBT experts in each respective country were asked to provide a narrative account of their country’s implementation journey. Histories were then considered in accordance with existing DBT implementation frameworks.

**Results:**

Common themes emerged such as the importance national guidelines on evidence-based treatment shape implementation, university affiliations promote dissemination, implementation and research activities, national societies help to create standards.

**Conclusion:**

There are common DBT implementation trajectories that exist across geography and nationally specific mental health care systems. The discussion of the refining of national DBT practices based on emerging literature and the importance of outcomes of implementation such as therapists’ behaviour or client related outcome data adds to existing information.

## Introduction

Over the last 40 years a revolution in the treatment of Borderline Personality Disorder (BPD) occurred. A significant aspect in the change of the landscape began with Marsha Linehan’s treatment Dialectical Behaviour Therapy (DBT). Linehan’s original aim was to create an effective treatment for individuals engaging in suicidal and self-harming behaviours [1, 2]. In the 1980s, to strengthen her applications for research funding, she incorporated the diagnosis of borderline personality disorder (BPD) into her work ([3], UW video lecture Dialectical Behavior Therapy (DBT): Where We Were, Where We Are and Where Are We Going). Publication of the treatment manual, Cognitive Behavioral Treatment for Borderline Personality Disorder, in 1993 further accelerated implementation efforts and firmly aligned DBT with the treatment of BPD.

Clinical implementation of DBT in Europe began around 1994, when Linehan provided training to a group of clinicians from the United Kingdom (UK), Germany, and the Netherlands. From that point, Germany, the UK and the Netherlands were the first European nations to establish robust DBT programmes, research initiatives, and national DBT societies. This paper reviews the implementation trajectories in five European countries, spanning over 30 years, and identifies both shared and divergent processes. The current research sits amongst literature about DBT implementation in systems as opposed to the work on implementing a single team. Common themes include the need for comprehensive training, an emphasis on adherence and fidelity to the treatment manual and structures, the benefits of research and treatment development and adaption, partnerships with universities, and the establishment of national DBT societies. While there are common themes, implementation processes remain highly specific to each country’s healthcare system.

### Implementation processes

Reviews by [4] and Navarro-Haro et al. [5] identify key facilitators and barriers to DBT implementation. One of the key factors in the implementation process is the presence of a DBT consultation team.

DBT’s team-based structure, considered essential by Linehan [1] to help increase therapist motivation and capacity to treat clients, focuses on reducing therapist burnout by providing a forum for therapists to use the tools of the treatment to attend to their own behaviors and reactions to the therapeutic process, and enhances delivery of the treatment with adherence and fidelity to model. Ultimately, the function of this mode is to create conditions that improve therapy for patients by maximising team-based efforts to enhance the treatment offered.

Many randomized controlled trials (RCTs) that demonstrate DBT’s efficacy and effectiveness have been delivered with the mode of consultation team model. More broadly, implementation literature indicates that team-based models are more likely to produce sustainable system-level change due to team processes [6, 7] and thus the team-based nature of the treatment may be account for part of the success of DBT. Nevertheless, data from a large scale real-world systemic implementation suggest that DBT has been delivered in large scale community-based implementations with varying degrees of fidelity to the model [8–11]. Additionally, some clinicians attempt to deliver DBT without a consultation team. Landes et al.,’s [11] study of the implementation of standard DBT across the Veterans Health Administration in USA by 59 teams reported that therapists on 26 teams either did not have a consultation team or that they did not really attend consultation team meetings.

Another key factor in effective DBT implementation in clinical settings is training of therapists from diverse professional group backgrounds (e.g., psychologists, psychotherapists, psychiatrists,, nurses, occupational therapists and social workers) and the preparation of the treatment setting or facility (planning of treatment components and treatment plans, spatial/infrastructural arrangements, reimbursement procedures with health insurance providers, etc.) [12]. Effective DBT implementation requires teams that are well-trained, sustainable, able to deliver comparable clinical outcomes, and responsive to the needs of the population [12, 13]. Reviews by [4] and Navarro-Haro et al. [5] describe implementation as beginning with a preparation phase shaped by organisational culture, communication practices, and information-gathering through external consultation. Organisational support, including protected time for clinicians to train and implement the model, and adequate funding, is critical to successful implementation. Creating an empowered implementation team that can design a plan aligned with both clinician interest and organisational priorities also enhances success.

DBT implementation can also be catalysed by broader systemic developments, such as legislative changes or shifts in accepted practices for treating individuals with severe and enduring mental health difficulties. Implementation is more likely to succeed when DBT’s structures, strategies, philosophy, and underlying assumptions help the host organisation fulfil its own values and goals. An early activity in the implementation process involves key stakeholders receiving an orientation to the treatment and engaging in discussions about the treatment’s fit with organisational aims. Such processes tend to strengthen organisational commitment to implementation. Establishing supportive organisational structures, such as job planning, communication pathways, accountability systems, financial resources, and physical space, increases the likelihood of long-term success. Additionally, it can help to orient team to information the likelihood staff turnover and the need to plan for relatively early in the implementation process to ensure that teams survive long-term [14].

The narrative research presented in this manuscript attempts to investigate the hypothesis that the DBT Implementation Framework (Tom et al., 2019) which integrates the stages of implementation discussed in wider implementation science research (planning, engaging, executing, evaluating and reflecting) with the processes that are unique to DBT implementation (context, DBT, evidence, and facilitation) in the establishment of new teams, may also provide a context for understanding longer terms national implementation processes. It also aims to examine whether national healthcare contexts and the time since the initial implementation of DBT may play a role, given that similar implementation outcomes have been achieved at roughly similar time points across these different countries.

### Methods

The national DBT journeys of the United Kingdom, Germany. Norway, Poland and the Czech Republic are described. These five countries were selected because they represent a wide range of time span since the initial implementation of DBT., with UK and Germany having first implemented DBT nearly 30 years ago and Norway approximately 20 years ago, Poland approximately 12 years ago and the Czech Republic approximately 8 years ago. These national implementations also present a mix of wealth and mental health care systems at various points of modernisation/reform. Each country selected to participate in the narrative analysis presented their national DBT journey a DBT dissemination conference organised by the research and implementation group of Project Gradient in the Czech Republic in June 2025. The selected countries represent early, mid, and recent implementers of DBT in change national health contexts. Each narrative description of the unique national history of DBT implementation is provided by a local DBT expert with knowledge of DBT’s developmental pathway in their respective country and is mostly presented as chronology of the nation’s implementation journey. Data sources included documentation from national societies as well as other relevant national policy documentation. Common themes were then highlighted by lead author in a table at the end of the results section. Themes were discussed in relation to existing implementation frameworks for DBT (Tom et al., 2019).

## Results case studies

Implementation trajectories in the UK, Germany, Norway, Poland and the Czech Republic are described one by one. Each author highlighted implementation themes that they considered most relevant for their respective country’s implementation as the national healthcare contexts shaping DBT implementation differed greatly. The nations presented their histories largely chronologically. Some common themes emerged and include the influence of public health initiatives, the development of training infrastructures, partnerships with universities, research initiatives and the establishment of independent professional organisations, which in two cases, the UK and Germany are responsible for assessing adherence and fidelity to the DBT model of therapists and teams. Each of these countries has a national DBT association that is a member of the European DBT Association. DBT work in these case study countries is transactionally constructing EDBTA processes, while simultaneously being shaped by them.

### The United Kingdom: the public health context and timeline of key developments in DBT

British Isles Dialectical Behaviour Therapy (biDBT), the most established DBT training organisation in the UK, estimates that slightly more than 250 teams currently deliver DBT in the UK. Over half operate in outpatient settings, and teams exist across the lifespan, with one-third working in child and adolescent mental health (CAMHS) and about 5% in older adult services. These teams offer either comprehensive DBT or “skills-only DBT,” also referred to as “all modes minus individual therapy.” DBT in the UK is provided in forensic settings, substance misuse, eating disorder services, used to treat post-traumatic stress disorder, as the RO-DBT adaptation, and in schools amongst other settings and populations. Reaching this level of provision has taken roughly three decades.

Implementation of DBT has been in part facilitated by changes in UK public policies. In the 1970s, psychological therapy in the UK increasingly emphasised evidence-based treatments (EBTs), marking a shift toward empirically supported interventions in mental health services. The 1990s saw the expansion of community-based care, promoting treatment outside institutional settings [15]. The establishment of National Institute for Clinical Excellence (NICE) in 2001 further embedded research-led practice by formally evaluating and endorsing evidence-based psychological interventions. In 2003, a key policy document stated that personality disorder (PD) should no longer be considered a diagnosis of exclusion [16]. This improved access to psychological treatment for people with PD and reduced structural stigma as the evidence base demonstrated BPD to be treatable. The 2003 publication also created a context in which a psychological therapy offered as a team, which was a departure from pervious provision of therapy, began to become an accepted method of treatment. In 2009, NICE published guidelines on treating BPD, designating DBT as the recommended treatment for women who engage in self-harm [17]. The synthesis of a decade of bottom-up implementation of DBT coupled with policy shifts created fertile conditions for acceptance of a team-based intervention and wider implementation of DBT in the UK.

### Development of DBT in the UK: A DBT training team with international affiliations

In 1994, Dr Michaela Swales, arguably the UK’s earliest DBT adopter, trained in the treatment with Professor Marsha Linehan in the United States. Upon returning to the UK, she established the first DBT programmes on an adolescent inpatient unit in Bangor, North Wales. Between 1994 and 1997, Swales, Dr Heidi Heard, a member of Marsha Linehan’s research team, and a colleague from the University of Leeds launched the first formal UK DBT training programme. Partnerships with universities can significantly support the implementation of emerging therapeutic models and research into their effectiveness.

A major milestone in long-term implementation was the creation of a national training infrastructure. biDBT, founded in 1997, maintained close ties with Linehan’s US-based training organisations and became the primary provider of DBT training in the UK and Ireland. biDBT offered evidence-based DBT training, including the 10-day Intensive Training™, which prepares whole teams to implement DBT through 5 days of instruction, followed by six months of self-directed implementation activities, and then another 5-day module focused on shaping clinical practice [18]. biDBT also delivered the 5-day Foundational Training for individuals joining established DBT teams [19].

### Research

Research conducted within the UK healthcare system further supported DBT’s dissemination. Two RCTs, Feigenbaum et al. [20] and Priebe et al. [21], evaluated DBT against treatment-as-usual in NHS adult outpatient settings. These studies, conducted in collaboration with University College London and Queen Mary University of London respectively, provided evidence for DBT’s acceptability and effectiveness in the UK context. The UK has produced a great deal of research on DBT often centred on aspects of implementation, qualitative studies as well as PhD dissertations and doctoral theses.

### Professional organisation: creating infrastructure and standards

Another milestone for the DBT community was the establishment of nationwide professional standards for DBT delivery through the creation of a professional society. The Society for Dialectical Behaviour Therapy in the UK and Ireland (SfDBT) [22], founded in 2012 by Dr Christine Dunkley and Professor Stephen Palmer, functions similarly to the British Association for Behavioural and Cognitive Psychotherapies (BABCP). An essential element in legitimising DBT was the professional society’s institutional independence from the leading UK training company, biDBT, and other training organisations. SfDBT hosts annual conferences and workshops to promote continuing professional development and networking.

In 2014, SfDBT introduced an accreditation process for DBT therapists, aligned with the Linehan Board of Certification (LBC) in the USA. Accreditation, more commonly known in other parts of the world as “certification”, requires clinicians to hold a core mental health professional qualification, complete 450 h of DBT training, be delivering comprehensive DBT, maintain an ongoing mindfulness practice, receive individual supervision, and submit work samples that are assessed for their adherence to the DBT model on the DBT-ACS [2] originally and more latterly the DBT-ACI [23] adherence rating measures used for individual therapy. The SfDBT UK and Ireland is also a member organisation of the European DBT Association (EDBTA).

SfDBT expanded its accreditation system in 2019 to include the accreditation of supervisors and, in 2021, began accrediting DBT training programmes. In 2024, SfDBT introduced accreditation for DBT treatment programmes, using the Program Fidelity Scale to assess team-level adherence [24].

### University degree

These efforts, similarly, to other implementation efforts, both influenced and were influenced by broader public health developments. In 2020, Bangor University and biDBT received NHS England funding to deliver a fully funded Postgraduate Diploma in DBT (https://www.england.nhs.uk/commissioning/spec-services/npc-crg/group-c/). The Post Graduate Diploma in DBT is a university degree equivalent to a taught masters’ degree without a thesis. The training specifications adopted by NHS England allowed for completers of the degree to have completed all of the SfDBT requirements for an accredited DBT Therapist. Ultimately, this course became an SfDBT level 3 accredited training course, which allowed for its graduates to be immediately accredited as DBT therapists (https://sfdbt.org/training-programme-accreditation/). Other level 1 and 2 university level DBT training courses have also started to appear in the UK.

### Benchmarking: moving from outputs to outcomes

As an extension of the Post Graduate Diploma, biDBT launched a national benchmarking project that includes a website to collect routine outcome data to support the evaluation and continuous improvement of DBT practice in the UK (https://dbt.uk.net/). Benchmarking allows teams offering either comprehensive DBT or “skills only” DBT to compare their programme outcomes with those of RCTS and peers. At a minimum, teams collect data at various points on client’s quality of life, difficulties in regulating emotion, and ways of coping. The data is immediately accessible to teams and individual therapists. Teams and therapists are encouraged to use the data to inform treatment discussions and consultation team meeting processes. Additionally, the website assists with work capacity calculations for teams and therapists. This system further moves the UK to a focus on outcomes. The movement from output to outcomes began with the introduction of therapist, supervisor, and programme accreditation and now the focus on client outcomes as well helps ensure that money invested in DBT delivers actual change clients’ behaviours and lives.

### Implementation pathways, and further developments of DBT in Germany

Dialectical Behaviour Therapy (DBT) was introduced in Germany in the mid-1990s and has since become one of the most widely implemented and empirically supported psychotherapeutic treatments for borderline personality disorder (BPD) and related high-risk clinical presentations. Its dissemination occurred within a healthcare system characterised by statutory health insurance coverage, a strong inpatient and psychosomatic treatment sector, and an established tradition of evidence-based psychotherapy. These structural conditions provided a favourable context for the “top-down” implementation of a comprehensive, team-based treatment model such as DBT, particularly for patients with severe emotion dysregulation, suicidality, and complex comorbidity.

### Early implementation and research foundations

A key driver of DBT dissemination in Germany was the early integration of clinical implementation with systematic research. In the mid-1990s, Professor Martin Bohus and colleagues at the Central Institute of Mental Health (CIMH) in Mannheim received direct training from Marsha Linehan and subsequently established one of the first comprehensive DBT programmes for patients with BPD in Germany [1]. From the outset, the Mannheim programme combined routine clinical delivery with rigorous outcome evaluation.

Early randomised controlled trials conducted in Germany demonstrated the effectiveness of DBT in reducing self-harm, suicidal behaviour, and psychiatric hospitalisation among patients with BPD when compared to treatment-as-usual [25, 26]. These studies contributed substantially to the international DBT evidence base and played a critical role in increasing the acceptability and feasibility of offering DBT within German psychiatry and psychosomatic medicine. Subsequent naturalistic and routine-care studies showed that DBT could be implemented effectively outside specialised research contexts, supporting its scalability within the German healthcare system [27].

### Training infrastructure and national dissemination

A defining feature of DBT implementation in Germany has been the early establishment of a nationally coordinated training and governance structure. Building on close collaboration with Linehan and US-based DBT training providers, German DBT leaders developed standardised, team-based training pathways emphasising supervised implementation, consultation teams, and long-term sustainability.

The Dachverband Dialektisch Behaviorale Therapie (DDBT), founded in the early 2000s, serves as the central professional organisation responsible for ensuring fidelity and high-quality implementation of DBT in Germany. The DDBT defines national training standards, specifies requirements for DBT team composition, and promotes continuing professional development through conferences, workshops, and supervision networks [28].

The German DBT dissemination system is characterised by a dual-structure model. Therapist certification is centrally regulated by the DDBT, whereas the delivery of DBT training is delegated to separate, franchise-licensed training institutions. This structural separation distinguishes certification governance from training provision and reflects a decentralised model of implementation support with centralised quality assurance.

Training is typically delivered in an intensive, team-based format comprising two multi-day didactic blocks, separated by an extended implementation phase in clinical practice. Foundational trainings are designed for clinicians joining established DBT teams, while advanced modules address specialised adaptations and further skill development.Consistent with Linehan’s original model, participation in a DBT consultation team is regarded as a core component of ethical and effective DBT delivery in Germany [1]. This strong emphasis on treatment adherence and fidelity has contributed to relatively consistent implementation standards across diverse clinical settings.

### Scope of clinical implementation

Over the past three decades, DBT has been implemented widely across Germany in psychiatric inpatient units, psychosomatic hospitals, day clinics, and outpatient services. Particularly strong uptake has occurred in specialised residential and day-treatment programmes for personality disorders and trauma-related conditions, where the intensity and structure of DBT align well with service configurations and reimbursement models.

DBT is explicitly referenced in German national clinical guidelines for the treatment of borderline personality disorder, reinforcing its status as a standard, evidence-based intervention within routine care [29]. Like developments in the UK and Norway, DBT dissemination in Germany has been facilitated by broader public-health shifts toward evidence-based practice and increased recognition of personality disorders as treatable conditions.

### Development and implementation of DBT for PTSD

One of the most influential contributions to the international DBT field emerging from Germany has been the development of DBT for Posttraumatic Stress Disorder (DBT-PTSD; [30]). This adaptation was developed at the CIMH to address individuals with PTSD related to childhood abuse who also present with severe emotion dysregulation, dissociation, and self-harm, patients frequently excluded from standard trauma, focused treatments.

DBT-PTSD integrates trauma-focused cognitive and exposure-based interventions within a DBT framework while retaining core principles such as hierarchical treatment targets, the balance of acceptance and change strategies, and therapist consultation teams. Treatment follows a structured, phase-based approach, with initial emphasis on safety and skills acquisition before the introduction of trauma processing [31].

A series of German randomised controlled trials demonstrated that DBT-PTSD is effective in reducing PTSD symptom severity, depressive symptoms, and global psychopathology in highly complex patient populations [30]. A large multicentre RCT with women with childhood abuse–associated PTSD who additionally met 3 or more DSM-5 criteria for BPD further showed that 1-year of outpatient DBT-PTSD treatment was superior to Cognitive Processing Therapy (CPT), with lower dropout rates and higher rates of symptomatic remission [32]. These findings supported the rapid implementation of DBT-PTSD in German inpatient and day-treatment settings and stimulated international dissemination.

### Further DBT adaptations in Germany

In parallel with DBT-PTSD, several additional DBT adaptations have been implemented and evaluated in Germany. DBT for adolescents (DBT-A), based on the original model by Rathus and Miller [33], has been widely adopted in child and adolescent psychiatric services. German implementations emphasise developmental considerations and caregiver involvement through multifamily skills groups. Empirical evidence from German-speaking contexts [34] and international trials indicates significant reductions in self-harm, suicidal ideation, and overall symptom severity [35].

Dialectical Behavior Therapy for substance use disorders (DBT-SUD) has been implemented in Germany primarily as DBT-S [36], a structured adaptation designed for patients with co-occurring borderline personality disorder and substance-related problems. DBT-S is now an established clinical option in specialized inpatient and outpatient settings. In Germany, the three main clinics offering comprehensive DBT-S program are: St. Hedwig-Krankenhaus (Alexianer) in Berlin, which provides the only certified inpatient DBT-S unit plus a day-clinic DBT-S-flex program for patients with borderline personality disorder and substance use disorders; the Central Institute of Mental Health (ZI) in Mannheim, which offers DBT-S as an established inpatient and outpatient treatment program for patients from across Germany; and the Karl-Jaspers-Klinik near Oldenburg, which runs a three-module inpatient DBT program that also addresses comorbid addiction and borderline pathology. The available German literature focuses largely on implementation, service delivery, and clinical feasibility rather than large local randomized efficacy trials; a nationwide retrospective survey of DBT service provision documented the limited availability of DBT slots, long waiting times, and gaps in outpatient care, which underscores the relevance of expanding DBT-S offerings but does not directly test efficacy for substance use outcomes [37]. Consequently, the strongest efficacy evidence for DBT in substance dependence remains the international randomized controlled trial data, particularly the studies by [38, 39], while German publications mainly document clinical adoption and the need for integrated treatment approaches rather than providing robust independent proof of superiority.

Dialectical Behavior Therapy for eating disorders (DBT-E) is an adaptation of standard DBT that was developed to address binge eating disorder, bulimia nervosa, and other eating disorders characterized by emotional dysregulation, with a primary focus on teaching skills for self-management of eating behavior, emotion regulation, and stress tolerance [40]. In Germany, DBT-ED is not implemented as a standardized, nationally manualised treatment programme. Instead, it is widely used as an integrative treatment approach within specialised services for eating disorders. In clinical practice, DBT-ED is typically embedded in psychosomatic, multimodal treatment programmes and combined with established evidence-based interventions. These usually include cognitive-behavioural therapy for eating disorders (CBT-E), DBT skills training focusing on emotion regulation and crisis management, and structured nutritional rehabilitation as part of a comprehensive treatment framework. In Germany, DBT-E has been implemented primarily in specialized eating disorder centers and psychiatric clinics, notably at the University of Lübeck (where DBT-E research and a pilot study are documented), the LVR-Klinikum Essen (which offers the certified DBT-A-ESS program for adolescents with eating disorders, including mindfulness skills, emotion regulation skills, interpersonal skills, self-esteem skills, distress tolerance skills, and family skills), and the Pfalzklinikum (which provides DBT-A for adolescents with eating disorders on 27 beds with a multidisciplinary team and has been DBT-certified since 2007) [41].

The scientific evidence base for DBT-E in German studies is limited to pilot studies and smaller clinical reports that suggest first promising efficacy in adolescent patients with anorexia and bulimia nervosa [41]. However, a standardized, nationally evaluated DBT-ED manual with randomized controlled trial evidence in Germany is still lacking.

DBT-ACES (Dialectical Behavior Therapy – Accepting the Challenges of Employment and Self-Sufficiency; [42, 43]) is an extension of standard DBT that was developed to support individuals with severe emotion dysregulation (including borderline personality disorder) after completion of standard DBT, with a particular focus on functional recovery and real-world self-sufficiency. The programme was conceptualised as a second-phase or “post-acute” DBT intervention rather than a crisis-stabilisation treatment.

Its central aim is to help clients achieve and maintain meaningful roles such as competitive employment, education, and independent living. To this end, DBT-ACES integrates standard DBT skills (emotion regulation, distress tolerance, interpersonal effectiveness) with additional behavioural and contingency-based strategies that directly target avoidance patterns and support goal attainment. A key structural feature is that treatment includes explicit, graduated expectations for engagement in work or vocational activity, alongside exposure-based and problem-solving interventions designed to reduce avoidance and increase behavioural activation toward life goals.

Empirical evidence for DBT-ACES is still limited but growing. Initial feasibility and observational studies suggest that DBT-ACES may improve vocational functioning and overall recovery outcomes in individuals who have completed standard DBT and continue to experience significant functional impairment. However, the evidence base remains smaller and less rigorous than for standard DBT, and DBT-ACES is generally considered an emerging, specialised adaptation rather than a fully established, widely implemented treatment model (Carmel et al., 2016; [43]) .

In implementation terms, DBT-ACES is often discussed as part of a broader “continuum of care” model in DBT dissemination, extending treatment focus from symptom stabilisation to long-term recovery, participation, and social reintegration.

### Development of DBT in Norway

#### Research as an implementation driver

Dialectical Behavior Therapy (DBT) was introduced in Norway at the University of Olso as part of the National Centre for Suicide Research and Prevention (NSSF) public health initiative to reduce self-harm and suicide amongst young people. The first formal training, led by Drs Alec Miller, as co-author of the evidence-based DBT-A adaptation [33], and Sarah Reynolds from the Behavioral Tech Institute (BTech), the USA based training company previously affiliated with Marsha Linehan, was organized by a clinical research team at NSSF in preparation for a randomized controlled trial (RCT) of DBT for adolescents with repeated self-harm. Clinical implementation of DBT was driven initially by a research agenda led by Professors Lars Mehlum and partially funded by the Norwegian Health Directorate and the DAM trust, a foundation that funds health and social welfare research. The first tranche of DBT teams consisted of research therapists at various sites within Oslo University Hospital. As is consistent with research, therapists on these teams received specialist training and supervision.

### Training team

Since 2006, the DBT Norway Training Program, operating as a university-affiliated non-profit within NSSF, has established a system for educating and developing therapists, supervisors, and trainers. The program developed nationally adapted guidelines for therapist education, trainer recruitment and development, and mentor/supervisor training. Teaching materials have been translated and adapted to Norwegian from English, and the center implemented a program for the continuing professional development of trainers and supervisors. Trainers can teach in both Norwegian and English.

### Professional standards

In many Norwegian health-care workplaces, competency is handled through internal training and approval and the continuing education requirements. There is a strong public sector, and in mental health services there is a preference for clear, regulated educational pathways (e.g. psychology degree + authorization). There is less tradition for independent therapist certification. Thus, the Norway DBT Training Program invests in a model of sustained, team-focused supervision with Team supervision over multiple years to many DBT teams across Norway, including opportunities to assess and address treatment adherence. Adherence rating materials have been translated into Norwegian in collaboration with the DBT-ACS [2] and DBT-ACI [23] calibration processes, however, there is not yet any formal process of certification of therapists. NSSF is in the process of converting its DBT training to a flipped-classroom model, increasing demands on pre-course preparation (e.g., learning objectives review, assigned readings, video lectures, short quizzes, literature summaries, and case-based question formulation). Didactic and demonstration videos have recently been produced to support this transition.

The Norwegian Intensive Training model follows the evidence based intensive training model [18]. Formal team and applicant requirements are explicit: teams should include at least four mental health practitioners, with a minimum of half being medical doctors or clinical psychologists. These criteria aim to promote patient safety, diagnostic competence, and the sustainability of local DBT services https://www.med.uio.no/klinmed/forskning/sentre/nssf/utdanning-kurs-konferanser/utdanning/dbt). Trainees complete assignments and examinations and implement DBT at their workplaces between the training weeks. New teams receive monthly consultation, and novice clinicians are mentored by experienced team members. Intensive training is provided both remotely and on-site. Foundational training for clinicians joining existing DBT teams consists of either five or ten full days [19]. NFFS also organizes advanced training courses and a biannual DBT conference. All NSSF trainers also function as supervisors, supplemented by two additional supervisory clinicians. The trainers offer supervision to DBT teams irrespective of experience level; currently, half of Norwegian DBT teams receive consultation from experts affiliated with the DBT Norway Training Program, and some teams have benefited from monthly supervision for many years.

The evidence-based implementation of DBT in Norway has led to research that demonstrated that DBT-A in Norway reduced self-harm in adolescents and increasing functioning, which was sustained into adulthood as evidenced by the 12 year follow up study [35, 44–46] and [47]. The strong focus on research is ongoing, and a research network with 8 collaborating units in Norway and Denmark is led by the center.

### Current DBT landscape in Norway

Currently, approximately 50 DBT consultation teams are established across all healthcare trusts in Norway, and more than 650 clinicians have been trained in DBT by the Norwegian DBT Training Team at the NSSF. The teams operate in a range of settings, including adult outpatient clinics, adolescent outpatient services, specialized services collaborating with child protection agencies, and a small number of substance misuse treatment units.

In 2025, the NSSF contributed to the development of new national guidelines for the treatment of personality disorders (https://www.helsedirektoratet.no/retningslinjer/personlighetsforstyrrelser-oppdagelse-utredning-og-behandling) [48]. A revised guideline, to be published in May 2026, lists DBT as one of the preferred treatment options. Recent developments include the implementation and adaptation of DBT-PTSD, DBT skills-only, DBT for ASD, Radically Open DBT, DBT group for parents/partners and DBT skills group used for substance use disorders (DBT-SUD). Furthermore, Intensive DBT is currently in pilot testing as preparation for a fully funded randomized controlled trial [49].

### Professional association

In 2017 a professional association, Norway-DBT, (N-DBT) aiming to spread knowledge, use and respect for DBT among clinicians, leaders, patients and their next of kin was established by Lars Mehlum. N-DBT is professional and developmental hub that supports DBT in Norway through: education, networking, research support, and implementation guidance across clinical services.

### Development of DBT in Poland: invalidating context

Poland provides a markedly different context for the implementation of DBT than the Western European and Scandinavian countries described above. There is virtually no institutional support for evidence-based psychotherapies, resources are limited, and university training in psychotherapy is rarely clinically oriented, while medical universities tend to marginalise psychosocial interventions as treatment options in favour of biological psychiatry. Most of the systemic facilitators of DBT implementation identified in the introduction to this paper, such as evidence-based mental health policy, research infrastructure, and clear institutional mandates, have been absent in Poland for many years and are unlikely to emerge soon. Instead, DBT has developed in Poland in the context of a broader crisis in psychiatry and mental health care, marked by chronic underfunding, political conflicts over how services should be organised, and the politicisation rather than professionalisation of key institutions. In this environment, clinical decision-making is rarely guided by evidence, international examples are not treated as models, and the mental health field is fragmented by long-standing tensions between professional groups and institutions. Despite the lack of facilitators and the presence of systemic obstacles, DBT has developed significantly in Poland over the last 10 years.

### Development of DBT in Poland: timeline and bottom-up approach

Over the past decade, DBT in Poland has developed almost entirely through bottom‑up initiatives. The Polish DBT Association (PTDBT) was founded in 2016 by a small group of clinicians who initially relied on self‑study, short international workshops, and online materials. In 2017 the first larger introductory training with Prof. Martin Bohus gathered around 100 participants, and in subsequent years 10 clinicians completed intensive training abroad and began working together in virtual consultation teams.

In 2018 the PTDBT organised the first international DBT conference in Poland. In the same year, cooperation with the National Educational Alliance for Borderline Personality Disorder (NEABPD) was initiated and the first online training for Family Connections (FC) programme leaders was conducted **(**https://www.neabpd.org/family-connections) [50]. At that point, it became apparent that there was very little institutional support or interest in DBT from academia, mental health authorities, or funding bodies in Poland.

Therefore, DBT for families in the form of the Family Connections programme, an evidence‑based, pro bono intervention delivered through volunteer networks worldwide, was the only realistic way to develop DBT‑informed services in Poland that matched the available bottom‑up pathway. Since then, around 200 12‑week FC groups have been delivered in Poland, reaching approximately 2,400 family members of people with BPD, emotion dysregulation, and suicidality. This became the primary way to reach the community, educate families about the effectiveness and evidence‑base of DBT. This encouraged a process of advocacy for appropriate care, which prompted many therapists to turn to DBT to better meet their patients’ needs.

### DBT training system in Poland

Since 2020, PTDBT has run an annual Comprehensive DBT training programme under the leadership of Prof. Alan Fruzzetti together with a gradually growing local training team. Each cohort completes four 3‑day modules over 9–10 months, combining didactic teaching with intensive skills practice, case discussions, and consultation‑team mentoring. Participants are organised into DBT teams, or encouraged to form them during the course, and are supported in implementing all treatment modes in their clinical settings as far as local conditions allow. The trainings have also centred on a transfer of technology with a gradual shift from Prof. Fruzzetti delivering the entire training to a shared format in which Polish trainers increasingly co‑teach and independently lead parts of the curriculum under ongoing supervision from Prof. Fruzzetti. The long-term aim is to develop an autonomous local training team.

At present, Poland has a small but clearly defined DBT community and infrastructure. Approximately 150 clinicians have completed Comprehensive DBT training, and there is a developing system of training and informal mentoring available in Polish. Around 20 teams currently offer some or all modes of a DBT programme, with about 10 private centres providing comprehensive, multi‑mode DBT services, several of which have proved sustainable over time. Key DBT manuals and books have been translated and are widely accessible, and introductory DBT and DBT skills modules have been incorporated into many 4‑year CBT training programmes. Family Connections groups are now available nationwide, including in several public institutions, with some groups co‑led by family members, so that DBT and FC increasingly provide both individual treatment and environmental interventions. In addition, PTDBT regularly organises national and international conferences and symposia with a recent milestone of this community‑driven development was the co‑organisation of the First European DBT Congress (EDBTA), held in Gdańsk in May 2025.

### DBT in Poland: challenges and dialectics

Despite these achievements, the development of DBT in Poland remains shaped far more by “in spite of” than “because of” systemic conditions. The clinicians most actively involved in DBT are almost all full‑time practitioners in the private sector, without access to protected research time or funding, which means that no systematic outcome studies of DBT have yet been conducted in Poland. Mental health authorities show little interest in promoting or commissioning evidence‑based treatments, and long‑standing tensions and political conflicts between institutions and professional groups further limit the chances of top‑down support. Against this backdrop, the Polish DBT story is less about how much could be achieved with favourable structures, and more about how much has been built through persistence, voluntary effort, and community collaboration in an invalidating system.

### Development of DBT in the Czech Republic

DBT has started to evolve considerably later in Czechia than in the European countries discussed above, with its early development taking place primarily outside the formal healthcare system. The first Czech DBT programme was established as a social service in 2008 at the therapeutic community Kaleidoskop (operating since 2006), led by Renata Tumlířová, which focused on individuals with various personality disorders. Members of this team were the first Czech workers trained in DBT at Behavioral Tech Institute in the USA.

### Early teams

For more than a decade, DBT development in the Czech Republic progressed slowly and remained limited to a small number of highly motivated teams in few mental health facilities in the country. Usually, only components of DBT were incorporated in otherwise treatment as usual practice. A significant milestone in the integration of DBT into the public healthcare system occurred in 2019, when following intensive training from biDBT in the UK, the first fully structured 24-week outpatient DBT programme was launched at the psychiatric clinic of University Hospital Brno led by Dr Pavla Horká Linhartová. This outpatient programme was fully funded by public health insurance and followed all standard DBT principles. Members of this team are also contributing to the growing local evidence base through early outcome studies focusing on the effectiveness of DBT for BPD in the Czech context [51], while also conducting wider research on BPD [7, 52].

Following this development, DBT services in the Czech Republic began to diversify in terms of settings and populations. In 2022, the first inpatient DBT programme for adolescents (14–17) was established at the Children’s Psychiatric Hospital in Opařany, incorporating intensive skills training, family involvement, and a structured daily programme.

### Research funding for additional team implementations

A further step toward a coordinated national implementation and outcomes evaluation was an initiative of the National Institute of Mental Health in Czechia, which has funded and coordinated the implementation of fully structured 24-week DBT programmes into 5 mental health facilities across multiple regions since 2023 as one of the goals of Project Gradient [53]. These teams were trained and supervised by biDBT. Large-scale dissemination and education are also an ongoing process that support the expansion of DBT components into various treatment programmes and facilities around Czechia, alongside increased availability of DBT programmes and other DBT-informed treatment for people with emotion dysregulation. These initiatives have been complemented by increased collaboration with European partners and participation in international training and research networks.

### Nascent professional organisation

The professional organisation of DBT practitioners in the Czech Republic remains in an early stage of development compared to countries with longer DBT traditions. In 2023, a dedicated DBT section was established within the Czech Association for Cognitive Behavioural Therapy (ČSKBT), providing an initial platform for networking, peer support, and discussion of therapy and training standards. These efforts are expected to result in the establishment of a national DBT association in near future, which will eventually provide a structure for therapist certification. Czech clinicians have also become increasingly active within the European DBT Association, presenting implementation processes and emerging clinical and research findings at international conferences and engaging with ongoing discussions around programme fidelity and adaptations.

Despite these advances, DBT availability in the Czech Republic remains limited in contrast to clinical need. Key challenges include a shortage of fully trained DBT teams and supervisors, and the nascent state of standardized national training formats and certification system aligned with international standards. Training for new teams as well as support of the stability of DBT teams by providing education to individuals filling in the gaps after staff turnover is a challenge. This gap led to the foundation of a private education institution Equilio – The Czech DBT Institute in 2026 by Dr Pavla Horká Linhartová and Martin Horký [54]. Additionally, another obstacle is funding, as reliance on project-based funding is not sustainable, and long-term solutions are needed. Coordination in such processes also needs an independent body of experts as part of its national association, to to set standards for evaluation of teams’ in adherence and fidelity to the model in accordance with international standards. At the same time, recent developments suggest a transition from isolated local initiatives toward more systematic, healthcare as well as social care-embedded implementation of DBT as a standard treatment for BPD in the Czech Republic.

### Summary of common themes by country

**Table 1:** Themes by country

Themes	UK	Germany	Norway	Poland	Czech Republic
Broader implementation context Public Health Drivers	Change in treatment of personality disorder	Focus on EBT and treatment of personality disorder	Plan to decrease self-harm and suicide amongst young people	Shift to post Soviet mental health system in national context	Shift to post Soviet mental health system in national context
Facilitator of implementation: Training Team	biDBT as most predominant in the UK and introduction of a university based Post Graduate Diploma in DBT	AWPs as training franchises granted from DDBT	Through University of Oslo	Through Polish DBT Association	Establised Equilio April 2026
Facilitator and evaluation of implementation: Research	2 RCTs, multiple other research projects related to implementation and PhD, Clinical Doctorate Projects	Rich research history on DBT as well as the biology of BPD. Research often published in German language. Research as driver for principle driven clinical adaptations e.g. DBT-PTSD/TF-DBT	Driver of initial implementaion of DBT. Large focus on research on DBT Adaptations e.g. DBT-A, DBT-SUD, DBT-PTSD	Not at present as local appetite for evidence-based practice not present	University Hospital Brno team, early adaptors, driving research. Also, outcomes on implementation reported internationally
Sustainability and evaluation of implementation Professional organisations All members of EDBTA	SfDBT in UK and Ireland: therapist, supervisor, programme and training accreditation	DDBT certifying therapists, supervisors, programmes, training	N-DBT without therapist certification	PTDBT without therapist certification	Czech Society for DBT without therapist certification
Outcome of impact of the implementation on the system Unique national contribution	Emphasis on implementation research. Shifting Focus on outcomes over outputs as end result of DBT implementation (Benchmarking)	Focus on treatment of BPD and CPTSD and principle driven treatment evolution: TF-DBT/DBT-PTSD	Importance of early intervention	Family systems work as a basis for bottom-up system change	Synthesizer of previous European implementations: focus on outcomes, biological research, incorporation of treatment adaptation

## Discussion

The DBT Implementation Framework (Tom et al., 2019) which integrates the stages of implementation discussed in wider implementation science research (planning, engaging, executing, evaluating and reflecting) with the processes that are unique to DBT implementation (context, DBT, evidence, and facilitation) focuses mostly the establishment of new DBT teams. The above case studies describe scaled up versions of DBT implementation processes in its description of five unique national DBT implementation pathways and is akin to processes described in the national implementation project in Ireland [55]. Apart from the situation in Poland, the implementation journeys described here are a mixture of bottom-up and top-down processes. These trajectories indicate that there are commonalities and some important differences related to system issues.

### Common facilitators: contextual factors and research as a driver for implementation and innovation

In all cases as we might expect from the DBT implementation literature context was key. Implementation processes began through the enthusiasm of one or two clinicians or researchers, which is a bottom-up factor that then transacted with and shaped top-down policies. Processes of DBT dissemination and implementation are facilitated by public policy support for evidence-based treatment and particular standards in mental health care. This was also seen in system-wide implementation projects such as the one in the USA Veterans Association [11]. Attention to the evidence-base also facilitates the incorporation of treatment adaptation as seen throughout Europe and particularly highlighted in the German system’s movement to the wide-spread dissemination DBT-PTSD. The pathway of implementation of DBT in the UK and in the Czech Republic has parallels in that they were both supported by wider systemic reforms of the mental health system to incorporate community care and evidence based treatments for people with severe and enduring mental health conditions such as BPD ([15], NHS 2003 & 2009; [56]). In the case of Poland, these missing elements have made implementation processes slower and arguably more arduous.

Additionally common themes such as access to quality, often evidence-based, training in DBT is highly valued. The importance of evidenced based training in general is widely known and has been shown to increase therapist experience and willingness to work with suicidal clients is a theme that has been explored in other implementation studies (e.g., [5]). University affiliation, which can be a driver for implementation, also increases the likelihood of research on DBT in the national environment being conducted, which was particularly observed in Norway. These examples also demonstrate that implementation is a long and dynamic process with common milestones such as the establishment of professional societies and/or the affiliation with professional CBT organisations or the establishment of research initiatives. Finally, local treatment landscapes such as a preference for inpatient or outpatient services, for example, in the case of Germany versus the UK, shape implementation processes.

The experience of implementation in Poland also demonstrates the benefit of ingenuity and commitment to learning in the realisation of ambitions to deliver evidence-based treatment. The Polish example further demonstrates another common theme, namely employing the strategies of the treatment itself and consider the system in which implementation is desired as the first DBT client can be effective [12]. Processes of system shaping are continuous and underpinned by the work of DBT Team leaders [12].

### Lessons for newly adapting countries

Harness the enthusiasm of local stakeholders and identify “DBT champions”, understood to be people in position of power who are enthusiastic and supportive of the implementation of DBT. DBT champions may not ultimately deliver DBT, and they can be powerful system influencers and on the other hand DBT champions may also be DBT Team leaders who both support the team internally and curate the interface of the team with the wider system. Where possible connect DBT with policies on mental health and the incorporation of evidence-based practice. Focus as quickly as possible on creating networks of interested practitioners both nationally through the creation of national societies or special interest groups in existing CBT professional organisations and internationally (e.g. EDBTA or WDBTA). Consider the indicators of success from the beginning. Plan to measure the impact of the implementation both by the establishment of a team with all functions and modes as well as by demonstrable changes in therapist and client behaviour through routine measuring of therapist adherence to the model in sessions and changes in client behaviours and quality of life.

## Strengths and limitations

In addition to providing information on common processes of DBT implementation and system change, this research adds to the DBT implementation framework literature by highlighting the importance of continuous adaptation of DBT provisions based on emerging research. Relatedly it also points to the need for constant evaluation of the clinical outputs from DBT implementation. Effective implementation pays attention changes in therapist (e.g. adherence and fidelity) and client behaviours (e.g. patient related outcome measures) and adjusts treatment offerings in accordance with data. This research also suggests that processes of therapist and programme certification as well as attention to client outcome through benchmarking activities are perhaps understudied aspects of the end processes of successful transfer of DBT from a lab based to a community-based activity. Even more data is needed to consider the interplay between client outcomes and therapist and team adherence and fidelity to the model. It also suggests that an examination of client outcome data can lead to treatment development such as in the Germany with the wide-spread evolution towards TF-DBT.

This narrative review relies on local DBT experts’ description of the implementation histories. Inevitably there will be recall biases and missing information. It is not designed to be comprehensive or even systematic put rather to provide broad descriptions.

## Conclusion

The implementation of DBT across Europe over the past 30 years has changed the treatment landscape for people with BPD. DBT has shaped and been shaped by changing mental health policy landscapes and treatment contexts. Discussion of multiple experiences at the national scale provides information for nations that are seeking to make DBT more widely available. Implementation is a dynamic, iterative, and continuous process of treatment refinement through the incorporation of emerging evidence on the increased effectiveness of treatment adaptations or modifications. Finally, this research also adds to existing literature this topic in its strong focus on post initial implementation processes.

## Data Availability

No datasets were generated or analysed during the current study.
